# Self-medication and knowledge of pregnant women about the use of medication during pregnancy in the cities of Rijeka and Zadar, Croatia

**DOI:** 10.3389/fphar.2025.1536050

**Published:** 2025-01-24

**Authors:** Željko Jovanović, Petra Vulić

**Affiliations:** ^1^ Faculty of Health Studies, University of Rijeka, Rijeka, Croatia; ^2^ General Hospital Zadar, Zadar, Croatia

**Keywords:** self-medication, pregnancy, women’s health, medication use, antenatal care

## Abstract

**Objectives:**

Pregnancy is a sensitive period during which the use of medicines, whether by prescription or self-medication, is a major challenge as it can have a significant impact on the health of the mother and the development of the foetus. It is important that pregnant women are well-informed about potential risks and benefits and seek advice from healthcare professionals before taking any medication, including over-the-counter medications, to ensure the safety and health of themselves and their unborn child. The aim of this study was to analyse the self-medication practices of pregnant women and their knowledge and attitudes toward medicines in the context of pregnancy. This includes analysing the way pregnant women make decisions about self-medication and their awareness of potential risks and benefits.

**Methods:**

A cross-sectional study was conducted on a sample of pregnant women from two cities of similar size and status, Zadar and Rijeka, in the period from 1 May 2023 to 1 June 2023 at the Clinical Hospital Centre Rijeka and the General Hospital Zadar.

**Results:**

Of the 128 women interviewed, 56% were taking prescription medication, most frequently in Rijeka, while 69.5% practised self-medication. The most commonly used medications were dietary supplements—75 (44.88%); over-the-counter pain relievers and fever, cough, allergy, cold, and nausea remedies—42 (33.07%); and herbal medicines—8 (6.29%).

**Conclusion:**

Pregnant women need to be better informed about self-medication and the safe use of medication. A total of 94.5% of respondents consider it important to improve educational programmes and access to information about the effects of medication on the health of the foetus and pregnancy outcomes.

## Introduction

Prescribing medicines during pregnancy is a major challenge for physicians, who must balance the potential benefits to the mother against the potential harm to the foetus. The dilemma is all the greater because, for many drugs, there are no relevant and absolutely certain data on their possible harmful effects during pregnancy. The available research shows that the majority of women take medication during pregnancy; 60% take prescription medication, 90% take over-the-counter medication, and 45% of pregnant women take various herbal preparations (Erdeljić Turk and Vitezić, 2017). It has also been observed that the perception of the risk of taking medication during pregnancy is high among both healthcare professionals and pregnant women, even for medications whose adverse effects have not been proven (Pole et al., 2000; Briggs et al., 2015; Food and Drug Administration, 2019; European Medicines Agency Guideline on Good Pharmacovigilance Practices, 2024). The percentage of women using over-the-counter medicines, herbal preparations, and dietary supplements is increasing (FDA, 2014). To address the need for updated pregnancy risk categories, the Food and Drug Administration (FDA) published a final rule in 2014. The FDA is amending its regulations governing the content and format of the “Pregnancy,” “Labor and delivery,” and “Nursing mothers” subsections of the “Use in Specific Populations” section of the label for human prescription drugs and biological products. The final rule requires the removal of pregnancy categories A, B, C, D, and X from the labelling of all human prescription drugs and biological products. The Pregnancy and Lactation Labelling Rule (PLLR) provides clear communication of available data to assist prescribers make critical decisions when treating pregnant or lactating women (FDA, 2014; Brucker and King, 2017; Byrne et al., 2020; Pernia and DeMaagd, 2016; Croatian Pharmaceutical Society, 2013). Self-medication is a form of treatment, in which the patient plays an active role, and it is increasing in manual sales in pharmacies due to faster and easier access to medicines that can be taken safely without medical supervision. It includes the selection and use of medicines by pregnant women to treat self-recognized conditions and symptoms. Self-medication products can be over-the-counter medicines, medical devices for self-medication, food supplements, special purpose cosmetics, other cosmetic products, general use items for self-treatment and prevention, herbal preparations, essential and herbal oils, homoeopathic remedies, and foods (FDA, 2014). Studies have shown that factors such as knowledge and health literacy, beliefs, or some socio-demographic characteristics, are associated with self-medication during pregnancy (Brucker and King, 2017; Byrne et al., 2020; Pernia and DeMaagd, 2016). Pregnant women who were more aware of the risks associated with self-medication are less likely to self-medicate than pregnant women with less knowledge. Some studies have shown that pregnant women with a university degree, with two or more pregnancies, and in the first trimester are more likely to self-medicate during pregnancy, mostly for practical reasons (Brucker and King, 2017). It has also been shown that pregnant women who are more aware of the risks of over-the-counter medicines are less likely to take them (Brucker and King, 2017).

For all these reasons, this study focused on three main objectives related to pregnant women’s behaviour and knowledge about self-medication and medication use during pregnancy. The first aim was to investigate how often pregnant women self-medicate without medical advice or recommendation. The second objective was to assess pregnant women’s knowledge of what medications can and cannot be taken during pregnancy, while the third objective was to examine the relationship between knowledge of medications intended for self-medication and the decision to take them.

## Subjects and methods

### Study design and setting

The study was conducted in a population of pregnant women for whom the entry criterion for participation in the study was adulthood, regardless of the current stage of pregnancy. The study was conducted on a sample of pregnant women from two cities of similar size and status, Zadar and Rijeka, Croatia, in the period from 1 May 2023 to 1 June 2023 at the Clinical Hospital Centre Rijeka and the General Hospital Zadar. A total of 128 women took part in the survey, of whom 78 (60.9%) were from Rijeka and 50 (39.1%) were from Zadar.

The survey was conducted by means of an anonymous online questionnaire created by the research authors using the Google Forms interface and distributed via social networks to all women who attended antenatal clinics during the data collection period. As it was a voluntary questionnaire, respondents completed it at their convenience and had the option to withdraw from the study at any time. The questionnaire consisted of 13 questions, which included questions on the demographic characteristics of the respondents, the use of medication during pregnancy, knowledge and attitudes towards the use of medication during pregnancy, and the need for and methods of education and information on the use of medication during pregnancy. The questionnaire took approximately 10 min to complete.

### Statistical analysis

Microsoft Office Excel was used for the statistical analysis of the data. The level of statistical significance for all tests carried out in this study was 0.05. Descriptive statistics were used to present the results of the questionnaire in the form of frequencies and relative proportions (percentages). Differences in the frequency of the attitudes and knowledge of the respondents and all hypotheses were analysed using the chi-squared test.

### Ethical aspects of the research

Prior to conducting the study, approval was obtained from the Ethics Committee of the Faculty of Health Studies of the University of Rijeka and the General Hospital of Zadar. Participation in the study was voluntary, with written informed consent. Before starting to complete the questionnaire, the respondents were introduced and the aim of the study and the method of completing the questionnaire were explained. Respondents completed the questionnaire independently, without coercion and with the option to withdraw at any time.

## Results

### Sociodemographic characteristics

A total of 128 pregnant women took part in the survey, of whom 78 (60.9%) were from Rijeka and 50 (39.1%) were from Zadar. Overall, the majority of the respondents, 69 (53.9%), were aged between 30 and 39, followed by 50 (39.1%) respondents aged between 21 and 29, while only 9 (7%) of the respondents were older than 40 years. In terms of educational level, the majority of the respondents had completed secondary education, namely, 52 (40.6%), or higher education, namely, 51 (39.8%), while only 13 (10.2%) respondents had completed postgraduate studies. In terms of stage of pregnancy, 82.8% of the respondents were at 21 weeks of gestation or more, while 17.2% were at 20 weeks of gestation or less. A total of 73 (57%) respondents were in their first pregnancy, while 55 (43%) were in their second pregnancy or more.

### Self-medication practice

**FIGURE 1:**
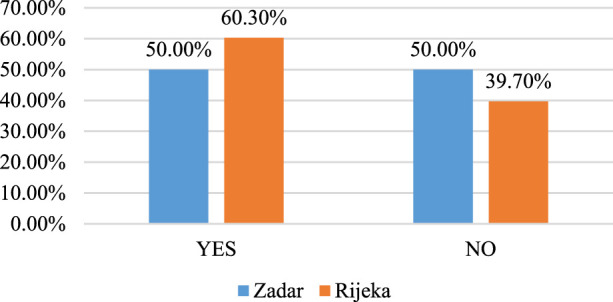
Use of prescription drugs.

**FIGURE 2:**
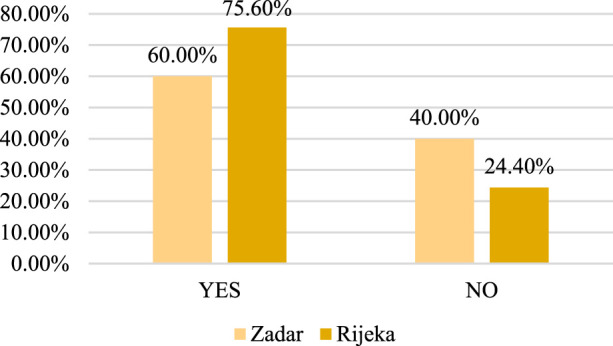
Frequency of self-medication.

The results show that 72 (56%) respondents are currently taking medication prescribed by a prescription doctor. The use of prescription drugs is shown in Figure 1, the list of the most frequently used drugs is shown in Table 1, and the frequency of self-medication is shown in Figure 2.

**TABLE 1:** List of the most commonly used prescription drugs.

Drug	Number of respondents
Levothyroxine	23 (38.98%)
Dydrogesterone	14 (23.72%)
Progesterone	10 (16.94%)
Iron supplements	6 (10.16%)
Folic acid supplements	3 (5.08%)
Alpha lipoic acid + magnesium and B-complex supplements	3 (5.08%)

The products and preparations most commonly used by respondents for self-medication are dietary supplements—75 (44.88%); over-the-counter pain relievers and fever, cough, allergy, cold, and nausea remedies—42 (33.07%); herbal medicines—8 (6.29%); and other medical products—2 (1.57%). Supplements included vitamins, minerals, omega-3 fatty acids, and other nutrients that are important for pregnant women’s health and proper foetal development. Herbal remedies, often containing natural extracts and herbal preparations, were used less frequently, but they were still present in the practice of self-medication by pregnant women: general supplements containing various vitamins and minerals—10 (13.33%), omega-3 fatty acids—23 (30.66%), probiotics—9 (12%), iron—24 (32%), and calcium—9 (12%). Over-the-counter medications included analgesics (paracetamol and ibuprofen)—12 (26.66%), for fever (paracetamol and ibuprofen)—18 (40%), for colds (phenylephrine, pseudoephedrine, and combination preparations)—8 (17.77%), and for nausea (meclizine and dimenhydrinate)—4 (8.88%). Of the herbal medicines, four pregnant women took echinacea and ginger. The most commonly used food supplements were, in particular, omega-3 fatty acids (30.66%) and iron (32%), indicating a high awareness of the importance of these nutrients for health, especially during pregnancy.

A summary of the responses to the questions about pregnant women’s knowledge about the use of medication during pregnancy is shown in Table 2. The level of knowledge was assessed based on the responses to questions on topics such as general attitudes towards the harmfulness of medicines for the foetus, the benefits of medicines for the health of the unborn child, the safety of using natural and herbal preparations and over-the-counter medicines for pregnant women and the foetus, the tendency of pregnant women to use natural and herbal preparations, the period of pregnancy when the use of medicines is most risky, and specific attitudes towards the safety of certain medicines during pregnancy. The overall level of knowledge could be rated good, with no statistically significant differences between districts, although there was still room for improvement.

**TABLE 2:** Pregnant women’s knowledge about taking medication during pregnancy.

	Zadar			Rijeka		
Question	Yes	I don’t know	No	Yes	I don’t know	No
All drugs are harmful to the child	2 (4%)	9 (18%)	39 (78%)	3 (9.84%)	6 (7.69%)	69 (88.46%)
Many unborn children are saved because mothers take medication during pregnancy when they are sick	26 (52%)	21 (42%)	3 (6%)	37 (47.43%)	29 (37.17%)	12 (15.38%)
Natural and herbal preparations can be used without danger to the pregnant woman and the foetus	6 (12%)	15 (30%)	29 (58%)	8 (10.25%)	19 (24.35%)	51 (65.38%)
Over-the-counter medications can be used without danger to the pregnant woman and the foetus	3 (6%)	9 (18%)	38 (76%)	6 (7.69%)	8 (10.25%)	64 (82.05%)
Pregnant women tend to use and take natural and herbal preparations during pregnancy	13 (26%)	29 (58%)	8 (16%)	34 (43.59%)	30 (38.46%)	14 (17.94%)
The most dangerous time to take all medications is in the first trimester	31 (62%)	14 (28%)	5 (10%)	51 (65.38%)	18 (23.07%)	9 (11.53%)
The riskiest period for the use of over-the-counter drugs is the first trimester	34 (68%)	12 (24%)	4 (8%)	52 (66.66%)	19 (24.35%)	7 (8.94%)
During the entire pregnancy, the painkiller ibuprofen can be used quite safely	2 (4%)	14 (28%)	34 (68%)	6 (7.69%)	20 (25.64%)	52 (66.66%)
During the entire pregnancy, the painkiller paracetamol can be used quite safely	37 (74%)	9 (18%)	4 (8%)	58 (74.35%)	13 (16.66%)	7 (8.97%)
Vitamin A-based acne medication (isotretinoin) is safe throughout pregnancy	0 (0%)	40 (80%)	10 (20%)	0 (0%)	52 (66.66%)	26 (33.34%)
Every pregnant woman should take folic acid	45 (90%)	2 (4%)	3 (6%)	72 (72.30%)	4 (5.12%)	2 (2.56%)
The use of omega-3 fatty acids is useful for the proper development of the foetus	36 (72%)	13 (26%)	1 (2%)	61 (78.20%)	15 (19.23%)	2 (2.56%)

The majority of pregnant women from both districts believed that not all medicines are harmful to the baby, which indicates that they were aware that taking some medicines during pregnancy could be safe, and this is confirmed by the fact that a considerable number of pregnant women from both districts responded that medicines can be the key to saving the life of the unborn child, especially if the mother is ill. There was also some uncertainty observed among pregnant women about the safety of natural and herbal preparations, with 24% of pregnant women from Rijeka and 30% from Zadar responding that they did not know whether such preparations are always safe during pregnancy. The majority of pregnant women, both in Rijeka (82%) and in Zadar (76%), believed that over-the-counter medicines are not always absolutely safe to use during pregnancy. The most dangerous time for taking any medications is the first trimester, as the vast majority of pregnant women in both cities know. Knowledge about the safety of certain medications such as ibuprofen and paracetamol during pregnancy was found to be not entirely satisfactory. Just as many pregnant women were aware that the use of ibuprofen during pregnancy is not safe (66%–68%), i.e., that paracetamol is the drug of choice as an analgesic during pregnancy, 26% of respondents did not have that knowledge. Although no one believed vitamin A-based acne medication (isotretinoin) to be safe during pregnancy, it is still worrying that 80% of pregnant women from Zadar and 67% of pregnant women from Rijeka did not know the answer to this question. A total of 73% of pregnant women from Rijeka and 90% of pregnant women from Zadar knew how important folic acid is during pregnancy, and the vast majority (72%–78%) believed that the intake of omega-3 fatty acids is beneficial for the proper development of the foetus. Almost all respondents, 94.5%, believed that pregnant women need to be more educated about self-medication and the impact of drugs, supplements, herbal preparations, etc., on the foetus and the pregnant woman. The majority of pregnant women expressed caution or uncertainty about the use of over-the-counter medicines, suggesting that they are aware of the potential risks and that they should consult professionals before taking such medicines. A considerable number of pregnant women believed that it is better for the foetus to avoid medications during pregnancy, even those that they would otherwise take. The claim that natural methods and herbal remedies can be used safely during pregnancy was met with great uncertainty and rejection, suggesting that better information is needed on this topic.

**FIGURE 3:**
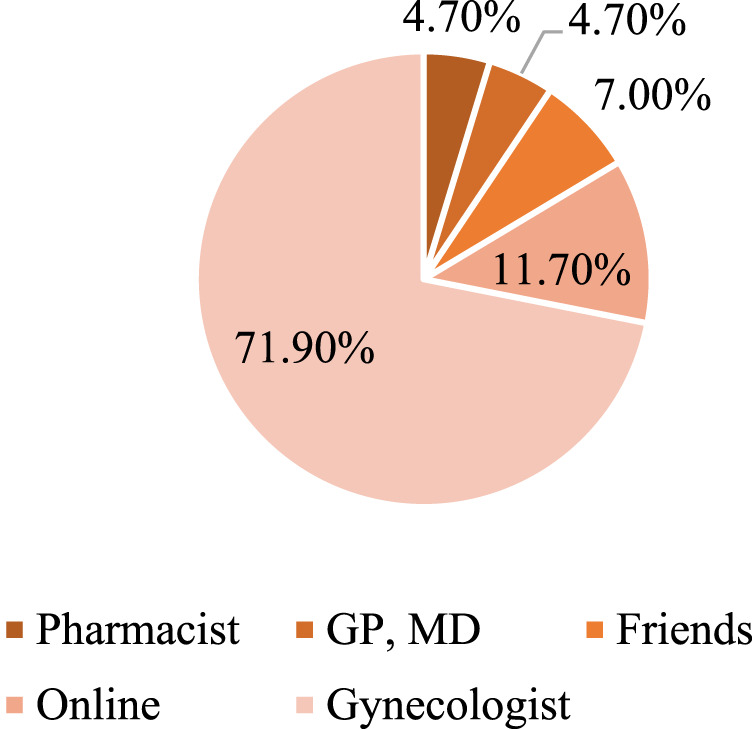
Sources of information about self-medication.

The majority of pregnant women believed that doctors prescribe too many medicines to pregnant women, suggesting an over-medicalisation of pregnancy, while 38% of pregnant women from Rijeka and 24% from Zadar believed that pharmacists have sufficient knowledge to advise on the safe use of medicines, supplements, and cosmetics during pregnancy. A summary of pregnant women’s attitudes towards taking medication during pregnancy can be found in Table 3, with attitudes being fairly similar in both cities. Figure 3 shows the most common sources of information for pregnant women. The majority of the respondents, 71.9% in total, declared seeking information from a gynaecologist, and 11.7% from the Internet and social networks. The importance of gaining information and counselling for pregnant women is shown by the answer to the last question as 94.5% of the respondents in both cities felt it was necessary to provide pregnant women and women with more information about self-medication and the impact of medications, food supplements, and herbal preparations during pregnancy and breastfeeding.

**TABLE 3:** Pregnant women’s attitudes about taking medication during pregnancy.

	Zadar			Rijeka		
Question	I agree	I’m not sure	I disagree	I agree	I’m not sure	I disagree
Over-the-counter medications are used to treat diseases that do not require a doctor’s visit	9 (18%)	7 (14%)	34 (68%)	12 (26.92%)	9 (11.53%)	57 (73.07%)
It is better for the foetus if a pregnant woman does not take medication during pregnancy, even if she would take the same medication if she was not pregnant	17 (34%)	18 (36%)	15 (30%)	25 (32.05%)	25 (32.05%)	28 (35.89%)
Natural methods of treatment and herbal remedies can be used without fear during pregnancy	2 (4%)	18 (36%)	30 (60%)	4 (5.12%)	19 (24.35%)	55 (70.51%)
Antibiotics can be purchased without a prescription	1 (2%)	1 (2%)	48 (96%)	1 (1.12%)	9 (11.53%)	68 (87.17%)
Vitamins can be purchased without a prescription	48 (96%)	2 (4%)	0 (0%)	75 (96.15%)	3 (3.84%)	0 (0%)
Pregnant women should consult a doctor when taking over-the-counter medications	50 (100%)	0 (0%)	0 (0%)	76 (97.43%)	1 (1.28%)	1 (1.28%)
Pregnant women should not take natural and herbal preparations without consulting a doctor	39 (78%)	5 (10%)	6 (12%)	63 (80.76%)	5 (6.41%)	10 (12.82%)
When a pregnant woman takes over-the-counter medications, there is a risk that it will harm the foetus	44 (88%)	3 (6%)	3 (6%)	66 (84.61%)	9 (11.53%)	3 (3.84%)
Over-the-counter medications are most often in oral form	18 (26%)	20 (40%)	12 (24%)	43 (55.12%)	23 (29.47%)	12 (15.38%)
Over-the-counter medications are most often in the form of gels or creams	2 (4%)	17 (34%)	31 (62%)	10 (12.82%)	26 (33.33%)	42 (53.84%)
The pharmacist has enough knowledge to be able to advise women on the safe use of medications, dietary supplements, and dermocosmetics during pregnancy	12 (24%)	19 (38%)	19 (38%)	30 (38.46%)	18 (23.07%)	30 (38.46%)
Doctors prescribe too many drugs to pregnant women	3 (6%)	18 (36%)	29 (58%)	9 (11.53%)	29 (37.17%)	40 (51.28%)

## Discussion

Among the 128 women who were interviewed, 56% took prescription medication, while 69.5% practised self-medication. The most commonly used medications were found to be dietary supplements—75 (44.88%), over-the-counter pain relievers and fever, cough, allergy, cold, and nausea remedies—42 (33.07%), and herbal medicines—8 (6.29%). Self-medication and the use of medication during pregnancy are issues of great importance. An objective problem related to medications and pregnancy is that the majority of women does not know about their pregnancy at the beginning and may be taking a potentially harmful medication during this time. According to some data from the literature, the majority of pregnant women take at least one medication during pregnancy, and the number of drugs used has increased over the last 30 years. Studies on medication use during pregnancy show that 80%–99% of pregnant women take medications, with an average of 4–7 drugs (Erdeljić Turk and Vitezić, 2017; Croatian Pharmaceutical Society, 2013; Atmadani et al., 2020; Vulić, 2023; Bohio et al., 2016; Beyene and Beza, 2018). Self-medication can be useful in many cases, but it is of particular concern in pregnant women, especially when advice from healthcare professionals is not sought or available. Such research has been conducted in Indonesia, for example, where access to health information may be even more limited (Atmadani et al., 2020). The results showed that there is a significant correlation between level of education and knowledge of self-medication. Pregnant women with higher levels of education are better informed about the potential risks and benefits of self-medication, while pregnant women with lower levels of education are more likely to self-medicate without being sufficiently informed about the potential risks (Atmadani et al., 2020). The results of other studies also point to the need to improve education programmes and access to information about self-medication, especially for pregnant women. Education on safe and effective self-medication should be integrated into the regular use of primary healthcare services during pregnancy and into counselling services for expectant mothers, including information on when self-medication is appropriate and which medications should be avoided during pregnancy (Vulić, 2023; Bohio et al., 2016; Beyene and Beza, 2018; Nkoka et al., 2018; Mbarambara et al., 2016; Ceulemans et al., 2019; Lupattelli et al., 2014; Hemaltha et al., 2013; Odalovic et al., 2012).

A multinational, cross-sectional, observational study involving 9,459 pregnant women from different parts of the world, including 286 pregnant women from Croatia, investigated factors associated with medication use during pregnancy. The study found that 81.2% of participants had taken at least one prescription or over-the-counter medication during pregnancy. Approximately 68.4% of pregnant women took medication to treat acute symptoms, and 17% took medication to treat chronic conditions (Lupattelli et al., 2014). There is undoubtedly a lack of literature on self-medication among pregnant women, not only in Croatia but in general. Our study also aimed to determine the prevalence of self-medication among pregnant women, the factors associated with it, the most commonly used drugs, knowledge and attitudes, and their impact on the practise of self-medication. Regarding the frequency of self-medication, in the two cities studied, the percentage of pregnant women who practise self-medication is statistically significantly higher than those who do not take over-the-counter preparations (χ^2 = 19.53, df = 1, *p* < 0.01). It can be concluded that self-medication is widespread among pregnant women in our study, which may have significant effects on their health and that of the unborn child if it is not in line with professional recommendations. A total of 89 of the 128 respondents (69.5%) stated that they used drugs, supplements, herbal medicines, or medical devices that were not prescribed by a doctor. The second aim of the study was to investigate the correlation between the level of education of pregnant women and their knowledge of medicines and their effects during pregnancy. The chi-squared test (χ^2 = 8.31, df = 3, *p* = 0.04) revealed a statistically significant correlation, suggesting that pregnant women with a higher level of education have a greater knowledge of medicines. The result supports our hypothesis and, thus, emphasises the importance of educating and informing pregnant women about drug use. The third aim related to the relationship between the level of knowledge about drugs and the frequency of self-medication, and the results of the chi-squared test (χ^2 = 1.55, df = 1, *p* = 0.213) showed no statistically significant relationship between these two variables. Therefore, the data do not support the hypothesis, which states that knowledge of medicines does not necessarily influence the frequency of self-medication in pregnant women. The study provides important insights into the behaviour and education of pregnant women in relation to drug use and self-medication. Although the results obtained and the research analyses conducted confirm that the majority of pregnant women self-medicates and that there is a statistically significant correlation between the level of education and knowledge about medication and its effects on pregnancy, it is somewhat surprising that no significant correlation was found between the level of knowledge about medication and the frequency of self-medication, indicating the need for better education of pregnant women about the safe use of medication. The analysis also shows the importance of educating pregnant women about the risks and safe use of medicines as self-medication is widespread and the level of education of respondents varies between regions (Hemaltha et al., 2013; Odalovic et al., 2012; Pereira et al., 2021; Jordan et al., 2022; Allegaert, 2022; Gerbier et al., 2022; Alsous et al., 2021; Coner, 2019; Alrabiah et al., 2017; Rahmani et al., 2019; Nawabi et al., 2021; Forghani et al., 2021). Also, in a Brazilian study on self-medication in pregnant women, the prevalence and associated factors, 107 (36.0%), of the 297 women surveyed had self-medicated in the previous 60 days. Paracetamol was the most commonly used medication, and headache was the most common symptom reported by self-medicating pregnant women (Pereira et al., 2021). Self-reported medication use among pregnant and postpartum women during the third wave of the COVID-19 pandemic in the European multinational cross-sectional study showed that a total of 2,158 of the 5,210 participants (41.4%) took at least one medication. Analgesics (paracetamol), systemic antihistamines (cetirizine), and drugs for stomach complaints (omeprazole) were the three most frequently used drugs. Anti-infectives were used less frequently than in the pre-pandemic period (Gerbier et al., 2022). The use of antidepressants and anti-anxiety medication remained similar despite the higher prevalence of these symptoms. Self-medication was found in 19.4% of women, and 4.1% of chronic drug users reported that they had changed their chronic medication of their own accord due to the pandemic. To summarise, the patterns of drug use in this study are broadly consistent with those of the first wave of COVID-19 and the pre-pandemic period. Further studies are needed to investigate the factors associated with self-medication and changes in chronic drug use due to the pandemic in the perinatal population (Gerbier et al., 2022). In a cross-sectional study of pregnant women in the northern region of Jordan involving 1, 313 pregnant women, self-medication and the use of herbal medicines were practised by 33.10% of the subjects, most commonly for headaches and pain in general (Alsous et al., 2021). On the other hand, in our survey, the majority of respondents (94.5%) believed that pregnant women need to be more educated about self-medication and the effects of medication on the foetus. This reflects similar concerns to previous surveys and emphasises the need for better education and information for pregnant women. A Croatian study on the characteristics of the use of over-the-counter medicines and supplements during pregnancy concluded that the majority of pregnant women in the study, 63.7%, do not self-medicate during pregnancy, and the main reason was the absence of symptoms that would require treatment with medicines or supplements. This answer was given by four out of five pregnant women surveyed. Other respondents stated that they could tolerate the symptoms they had without self-medication, while some chose not to take medication for fear of side effects or in the belief that the medication would not be effective or would be harmful to the baby. The results suggest that pregnant women are becoming increasingly aware of the risks of medication due to easier access to information. The majority of pregnant women did not agree with the idea of avoiding medication altogether during pregnancy, and almost 82% of them considered the first trimester to be the riskiest time for medication use. The majority of pregnant women recognised the importance of taking medications for healing during pregnancy and the use of prenatal supplements for the proper development of the foetus. Opinions on the use of paracetamol and ibuprofen are divided, with slightly more uncertainty about ibuprofen (Coner, 2019). According to the results of a previous study, the most commonly used drugs during pregnancy are analgesics, drugs for irritation and pain in the throat and pharynx, decongestants, and antacids, followed by combination preparations for colds, drugs for constipation, and antihistamines (Vulić, 2023). In addition, a significant percentage of pregnant women in Croatia reported using vitamins and minerals, herbal teas, other dietary supplements, probiotics, essential and herbal oils, herbal medicines, and homoeopathic remedies (Vulić, 2023). Comparing this with the results of this study, a significant majority (69.5%) used drugs, supplements, or over-the-counter herbal remedies, indicating a widespread practice of self-medication among pregnant women. The results show that attitudes and knowledge about self-medication vary among pregnant women, with some uncertainties and inconsistencies regarding the use of certain drugs and supplements, such as the warning of insufficient knowledge about the teratogenicity of isotretinoin for acne treatment. It can therefore be concluded that, despite the good level of knowledge among pregnant women, there is still a considerable need for better education and information, particularly in relation to safe self-medication. This is consistent with the results of previous studies that have highlighted the need for better education programmes and better access to information, especially in the context of specific local conditions, such as in less developed areas of the world such as Malangu in Indonesia, Iran, or Ethiopia, along with studies from Canada, Italy, Serbia, and other European countries (Atmadani et al., 2020; Vulić, 2023; Bohio et al., 2016; Beyene and Beza, 2018; Odalovic et al., 2012; Pereira et al., 2021; Jordan et al., 2022; Allegaert, 2022; Gerbier et al., 2022; Alsous et al., 2021; Coner, 2019; Alrabiah et al., 2017; Rahmani et al., 2019).

The survey points to the importance of better education and information for pregnant women on the safe use of medicines, with particular attention to differences in education levels and geographical areas. In addition to doctors, nurses, and midwives, pharmacists also play an important role. The counselling role of the pharmacist is extremely important. The pharmacist is the last member of the healthcare team that a pregnant woman comes into contact with before taking a medicine and also the one who is most easily accessible in the healthcare system. Therefore, the pharmacist is in the best position to provide professional advice to the patient, even if this study expresses a certain mistrust of pharmacists, which should certainly be changed through certain public health campaigns. Evidence-based education of pregnant women by a healthcare professional and a frank discussion with a doctor, nurse, midwife, or pharmacist is the most effective way to avoid possible health risks to mother and child or undesirable consequences of treatment or self-medication. Health literacy is a public health priority around the world, including for women from an early age, in order to maintain and improve sexual and reproductive health (Nawabi et al., 2021; Forghani et al., 2021).

Limitations of this study were the total number of respondents and the limitation to only two cities, which may not be representative enough to generalise the results to the total population of pregnant women in Croatia; however, this is a good pilot project for the continuation of the research on this topic. The survey relies on self-reporting by the respondents, which may lead to biases such as social desirability or unconscious distortion of information. Inconsistencies in age groups and education levels may influence the results and their interpretation. The study focussed on specific categories of medicines and may not have fully investigated the use of, or attitudes towards the wide range of medicines and therapeutic methods available to pregnant women. However, these shortcomings do not detract from the importance of the work and the results obtained, which can be compared with similar studies in the literature. Health literacy is a prerequisite for better health, especially during pregnancy and breastfeeding, where university-trained midwives can play a special role alongside doctors or pharmacists, which should be utilised more in the future when conducting public health campaigns on maternity protection.

## Conclusion

Self-medication and medication use during pregnancy require special care due to the particular challenges and risks associated with pregnancy. Pregnant women should take extra care and consult a doctor, nurse, midwife, or pharmacist before taking any medication, even those available over the counter. Research shows the importance of educating pregnant women about the potential risks of self-medication and the safe use of medication. The education of pregnant women about self-medication and the safe use of medication needs to be improved because 94.5% of the respondents considered it important to improve the quality of education programmes and access to information regarding the use of medicines during pregnancy.

The availability of reliable information and advice needs to be improved to enable pregnant women to make informed health decisions that benefit their health, the health of the foetus, and pregnancy outcomes.

## Data Availability

The raw data supporting the conclusions of this article will be made available by the authors, without undue reservation.
